# TACC3 Is Essential for EGF-Mediated EMT in Cervical Cancer

**DOI:** 10.1371/journal.pone.0070353

**Published:** 2013-08-01

**Authors:** Geun-Hyoung Ha, Jung-Lye Kim, Eun-Kyoung Yim Breuer

**Affiliations:** Department of Radiation Oncology, Stritch School of Medicine, Loyola University Chicago, Maywood, Illinois, United States of America; II Università di Napoli, Italy

## Abstract

The third member of transforming acidic coiled-coil protein (TACC) family, TACC3, has been shown to be an important player in the regulation of centrosome/microtubule dynamics during mitosis and found to be deregulated in a variety of human malignancies. Our previous studies have suggested that TACC3 may be involved in cervical cancer progression and chemoresistance, and its overexpression can induce epithelial-mesenchymal transition (EMT) by activating the phosphatidylinositol 3-kinase (PI3K)/Akt and extracellular signal-regulated protein kinases (ERKs) signal transduction pathways. However, the upstream mechanisms of TACC3-mediated EMT and its functional/clinical importance in human cervical cancer remain elusive. Epidermal growth factor (EGF) has been shown to be a potent inducer of EMT in cervical cancer and associated with tumor invasion and metastasis. In this study, we found that TACC3 is overexpressed in cervical cancer and can be induced upon EGF stimulation. The induction of TACC3 by EGF is dependent on the tyrosine kinase activity of the EGF receptor (EGFR). Intriguingly, depletion of TACC3 abolishes EGF-mediated EMT, suggesting that TACC3 is required for EGF/EGFR-driven EMT process. Moreover, Snail, a key player in EGF-mediated EMT, is found to be correlated with the expression of TACC3 in cervical cancer. Collectively, our study highlights a novel function for TACC3 in EGF-mediated EMT process and suggests that targeting of TACC3 may be an attractive strategy to treat cervical cancers driven by EGF/EGFR signaling pathways.

## Introduction

Epithelial-mensenchymal transition (EMT) is a highly conserved biological process that results in a conversion of polarized epithelial cells to mesenchymal cell types characterized by the loss of E-cadherin-mediated cell-cell contacts as well as the acquisition of increased migratory and invasive potentials [1]–[9]. Transcriptional factors Snail, Slug, Twist and Zeb1 have been identified as negative regulators of E-cadherin and are considered to be potent oncogenic inducers of EMT [10]–[14].

Despite the great success of early screening programs, cervical cancer is still the leading cause of gynecological death among women worldwide [15], [16]. Human papillomaviruses (HPVs) are thought to be the main cause of cervical cancer; however, studies have shown that the virus alone is not enough to develop cancer [15], [17], [18]. Epidermal growth factor (EGF) has been shown to be one of the most potent inducers of EMT in cervical cancer and associated with cervical stromal invasion and nodal metastasis [15], [19]. Chronic EGF treatment induces EMT via up-regulation of EMT-inducing transcription factor Snail in cervical cancer cells, and EGF-mediated EMT is correlated with EGF receptor (EGFR) overexpression and clinical progression of cervical cancer [15], [20]. The expression of EGFR has been found to be overexpressed in cervical cancer [21].

The transforming acidic coiled-coil protein (TACC) family is characterized by a conserved C-terminal “TACC domain”, essential for the interaction with tubulin and microtubules [22]–[24] and has been known to play a key role in the regulation of centrosome and microtubule dynamics [22], [25]–[29]. There are three TACC proteins identified in human: TACC1, TACC2 and TACC3 [24], [30]–[32]. TACC3 is involved in the assembly and organization of microtubules and chromosome alignment during mitosis, the maintenance of nuclear envelope structure and the regulation of cell growth/differentiation and gene transcription [24], [26], [27], [29], [33]–[38]. Depletion of *TACC3* causes growth retardation and embryonic lethality in mice due to increased apoptosis [39].

Although the role of TACC3 in human cancer is not clear, mounting evidence suggests that deregulation of TACC3 may be directly or indirectly linked to certain types of human cancer [24]. Genetic variations on chromosome 4p16.3, the region encoding *TACC3*, are found in various human cancers [9], [24], [40]–[48]. The fibroblast growth factor receptor 3 gene (*FGFR3*) and *TACC3* are closely localized on chromosome 4p16.3 [9], [32]. Recently, a TACC3-FGFR3 fusion protein was reported in a subset of glioblastoma multiforme (GBM) [49] and bladder tumor tissues and cell lines [50]. This fusion protein induces mitotic defects and aneuploidy and activates mitogen-activated protein kinase (MAPK) signaling pathway [49], [50]. So far, a somatic mutation (E680K) and two constitutional mutations (S93L and G514E) of *TACC3* have been identified in GBM and ovarian cancer, respectively [40], [51], [52]. Studies have shown that up-regulation of TACC3 is found in glioblastoma, non-small cell lung cancer (NSCLC) and multiple myeloma [40], [53], [54] and may contribute to lymphomagenesis [55], [56]. Gene expression profiling analysis has revealed that *TACC3* is up-regulated during the transition of ductal carcinoma *in situ* to invasive carcinoma of the breast and in ovarian cancer [57]–[59]. We have previously proposed that TACC3 may be directly or indirectly associated with tumor progression and drug resistance of cervical cancer, based upon data acquired from microarray analysis to identify genes regulated by TACC3 [24], [60]. Moreover, our recent study has shown that ectopic expression of TACC3 enhances proliferation, migratory/invasive ability and transformation capacity of HeLa cervical cancer cells and displays a more mesenchymal phenotype, accompanied by down-regulation of epithelial marker E-cadherin and up-regulation of mesenchymal markers N-cadherin and Vimentin as well as EMT inducers Snail and Slug [9]. On the other hand, depletion of TACC3 is capable of reversing/suppressing EMT [9]. Although our finding indicates that TACC3 may play an important role in EMT, the upstream signaling events responsible for TACC3-mediated EMT remain to be determined.

Herein, we demonstrate that TACC3 is overexpressed in cervical cancer. TACC3 can be induced by EGF, and EGF-mediated TACC3 induction is dependent on EGFR activation. Importantly, in the absence of TACC3, EGF is not able to induce EMT, suggesting that TACC3 is necessary for EGF-mediated EMT in cervical cancer. Moreover, we find a correlation between TACC3 and EGF inducer Snail in cervical cancer. Our findings, therefore, identify a novel mechanism that mediates EGF/EGFR-induced EMT and a potential therapeutic target for cervical cancer.

## Materials and Methods

### Tissue Microarrays and Immunohistochemistry

Cervical cancer tissue microarrays (CR805, CR1003 and CR1501) were purchased from US Biomax (Rockville, MD). Tissue microarray patient information is shown in Table 1. Tissue microarray slides were deparaffinized, rehydrated and heat-treated for antigen retrieval prior to antibody staining [61]. The slides were incubated with an anti-TACC3 antibody (Santa Cruz Biotechnology, Santa Cruz, CA) for 1 h at room temperature, followed by incubation with secondary biotinylated antibody and the Avidin Biotin complex (ABC) in accordance with the VECTASTAIN ABC kit protocol (Vector Laboratories, Burlingame, CA). After developing color with diaminobenzidine (DAB), the slides were independently assessed by authors. The intensity of staining was recorded as follows: 0 for negative expression; 1+ for weakly positive expression; 2+ for medium positive expression; and 3+ for highly positive expression. Photomicrograph (magnification ×100) was taken by DP12 microscope (Olympus, Tokyo, Japan) equipped with DP71 digital imaging system (Olympus).

**Table 1:** Cervical cancer tissue microarray information.

CR805		CR1003		CR1501	
Variable	n	Variable	n	Variable	n
**Age (years)**		**Age (years)**		**Age (years)**	
<50	56	<50	56	<50	84
≥50	24	≥50	44	≥50	66
**FIGO stage**		**FIGO stage**		**FIGO stage**	
Ia	7	Ia	48	I	68
Ib	15	Ib	10	Ib	40
IIa	3	II	10	Ic	2
IIb	17	III	2	II	14
IIIa	4	IIIb	10	IIa	6
IIIb	24			IIb	6
				IIIb	4
**Grade**		**Grade**		**Grade**	
1	32	2	25	1	4
2	66	3	55	1–2	1
3	19			2	43
				3	19

### Cell Culture, Antibodies and Reagents

The human cervical cancer (HeLa, CaSki, SiHa and C33A) and HPV-immortalized ectocervical epithelium (Ect1/E6E7) cell lines were purchased from the American Type Culture Collection (ATCC) (Manassas, VA). HeLa cells were grown in Dulbecco's Modified Eagle Medium (DMEM) (HyClone, Logan, UT) supplemented with 10% fetal bovine serum (FBS) (Gemini Bio-Products, Woodland, CA) and 1% penicillin/streptomycin solution (Thermo Fisher Scientific, Waltham, MA). CaSki cells were maintained in RPMI-1640 medium (HyClone) containing 10% FBS and 1% penicillin/streptomycin solution. SiHa and C33A cells were grown in MEM (HyClone) supplemented with 10% FBS and 1% penicillin/streptomycin solution. Ect1/E6E7 cells were grown in Keratinocyte-Serum Free medium (Ker-SFM; GIBCO/BRL Life Technologies, Gaithersburg, MD) with 0.1 ng/ml human recombinant EGF, 0.05 mg/ml bovine pituitary extract and additional calcium chloride 44.1 mg/L (final concentration 0.4 mM). Cells were incubated at 37°C in a humidified incubator with 5% CO_2_. Anti-TACC3 antibody was purchased from Santa Cruz Biotechnology. Antibodies against E-cadherin, N-cadherin, Vimentin, Snail, Slug and EGFR were purchased from Cell Signaling Technology (Beverly, MA). Anti-β-actin antibody and EGF were purchased from Sigma (St. Louis, MO). The EGFR kinase inhibitor AG1478 was purchased from Selleckchem (Houston, TX).

### shRNA and Transfection

Cells were transfected with a TACC3-specific or a control shRNA (Santa Cruz Biotechnology) by using FuGENE 6 (Roche Molecular Biochemicals, Indianapolis, IN) according to the manufacturer’s instructions.

### Western Blot Analysis

Human normal cervix tissue lysates were purchased from Imgenex (San Diego, CA). Cell extracts were prepared in a lysis buffer consisting of 50 mM Tris-Cl (pH 7.4), 1% nonyl phenoxypolyethoxylethanol (NP-40), 0.25% sodium deoxycholate, 150 mM sodium chloride (NaCl), 1 mM ethylenediaminetetraacetic acid (EDTA), 1 mM phenylmethylsulfonyl fluoride (PMSF), 1 mM sodium fluoride (NaF) and Complete protease inhibitor cocktails (Roche Molecular Biochemicals). Cell lysates were cleared by centrifugation at 13,000 rpm for 10 min, and the supernatants were subjected to western blot analysis. Equal amounts of protein were separated by sodium dodecyl sulfate (SDS)-polyacrylamide gel electrophoresis (PAGE) and transferred to nitrocellulose membrane. After blocking with Tris-buffered saline (TBS)/0.1% Tween 20 (TBS-T) supplemented with 5% nonfat dry milk for 1 h, membranes were incubated with primary antibodies diluted in blocking buffer for 2 h at room temperature or overnight at 4°C, followed by incubation with horseradish peroxidase (HRP)-conjugated secondary antibodies (Bio-Rad, Hercules, CA) for 1 h at room temperature. Finally, antigen-antibody complexes were detected by the enhanced chemiluminescence (GE Healthcare, Buckinghamshire, UK). Bands were quantified using imageJ software (http://rsb.info.nih.gov/ij/, NIH, Bethesda, MD) and normalized to β-actin.

### Quantitative Real Time-polymerase Chain Reaction (qRT-PCR)

Total RNAs from cells were extracted using the RNeasy® mini kit (Qiagen Sciences, Germantown, MD) and then subjected to cDNA synthesis using the RevertAid ™ First Strand cDNA Synthesis Kit (Fermentas Life Sciences Europe, Bremen, Germany). Expression of genes was analysed using an iQ™ SYBR^R^ Green supermix (Bio-Rad) and an iCycler iQ5 real-time PCR detection system (Bio-Rad). The following cycling conditions were used : 95°C for 5 min and 30 sec, followed by 40 cycles of 15 sec at 95°C and 1 min at 60°C. Data was analyzed with a normalized gene expression method (ΔΔ Ct) [62] using the iQ5 Optical System Software (Bio-Rad), and the *β-actin* was used as a reference for normalization. The sequences of the primer pairs were as follows: *TACC3* 5′-gaactggggaagatcatgga-3′ and 5′-ctcttcgttcttgcggtagc-3′ [63]; *β-actin* 5actinINK \l \o “Ulisse, 2007 #484” gtagc-3′ cgg-3i [64]. All the measurements were performed in triplicate.

### Transwell Migration Assay

Uncoated cell culture inserts (8-µm pores, 24-well) (Greiner Bio-One, Monroe, NC) were seeded with 1×10^5^ cells in 200 µl of serum-free media. The lower chambers were filled with 750 µl of complete media containing 10% FBS. After 16 h of incubation, cells on the upper surface of the filter were wiped off, and cells that had migrated to the lower surface of the filter were fixed with 4% paraformaldehyde or ice cold methanol for 5 min, stained with 0.05% crystal violet for 20 min and quantified spectrophotometrically at 490 nm.

### Invasion Assay

Invasion assays were performed using the QCM ECMatrix cell invasion assay kits (Millipore, Temecula, CA) according to the manufacturer’s instructions.

### Statistical Analysis

Statistical analyses were performed using GraphPad Prism 5.0 (GraphPad, San Diego, CA). Significance was determined by *t*-test and one-way Analysis of Variance (ANOVA), followed by the Tukey multiple comparison test. Pearson's correlation coefficient was used to measure the correlation between two variables. A *p-value* of less than 0.05 was considered as statistical significance.

## Results

### TACC3 is Overexpressed in Cervical Cancer

**Figure 1:**
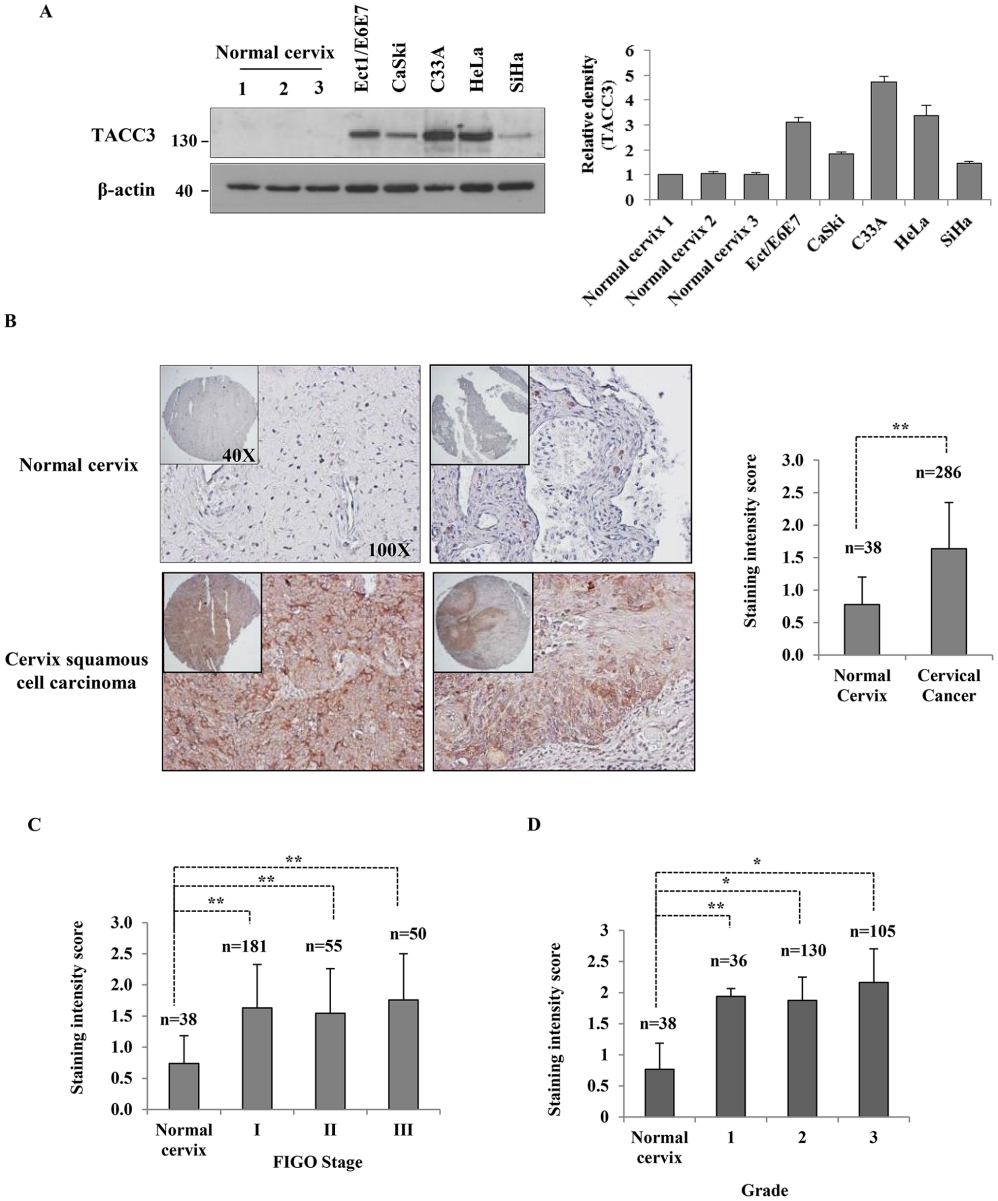
TACC3 is overexpressed in cervical cancer cell lines and tissues. (**A**) The expression of TACC3 in Ect1/E6E7 (HPV-immortalized ectocervical epithelial), CaSki (HPV-16), C33A (HPV-negative), SiHa (HPV-16) and HeLa (HPV-18) cell lines was determined by western blot analysis. The expression levels were compared to three normal cervix tissues. β-actin was used as a loading control. The intensity of bands was quantified using imageJ software and normalized to β-actin. Data shown are means ± SD of at least three independent experiments. (**B**) Representative immunohistochemical staining on cervical cancer tissue microarray. Quantitative analysis of cervical cancer tissue microarrays showed that the expression of TACC3 is higher in cervical cancer than in normal cervix, but its expression does not correlate with tumor stage (**C**) or grade (**D**). Data shown are means ± SD of at least three independent experiments. *, *p*<0.05; **, *p*<0.001.

To investigate the clinical importance of TACC3 in human cervical cancer, we first examined the expression of TACC3 mRNA in cervical cancer using the publicly available Oncomine database (www.oncomine.org, Compendia Bioscience, Inc., Ann Arbor, MI) [65] and determined that TACC3 is highly expressed in cervical cancer [66], [67]. Overexpression of TACC3 in cervical cancer was further confirmed in several cervical cell lines, including Ect1/E6E7 (HPV-16 E6/E7 transformed ectocervical epithelium), CaSki (HPV-16), C33A (HPV-negative), HeLa (HPV-18) and SiHa (HPV-16) compared with three normal human cervix tissues. As shown in Figure 1A, the expression of TACC3 in these cell lines was higher than that of normal cervix tissues. Interestingly, there was no significant difference in the expression of TACC3 between HPV-negative C33A and other cells carrying HPV oncogenes (Ect1/E6E7, CaSki, HeLa and SiHa), suggesting that HPV infection may not be responsible for the overexpression of TACC3. We also immunohistochemically analyzed the expression of TACC3 using cervical cancer tissue microarrays. TACC3 was almost undetectable in normal cervix, whereas its strong expression was observed in cervical cancer tissues (Figure 1B). However, there was no significant association of TACC3 expression with clinical stage or grade of the disease (Figure 1C, 1D and S1). Based upon our findings that the expression of TACC3 is elevated in cervical cancer compared to normal cervix but not significantly associated with disease progression, we suggest that increased expression of TACC3 may occur in the early stages of tumor development as well as be essential in maintaining cervical tumorigenesis.

### EGF Stimulation Induces Endogenous TACC3 Expression in EGFR-expressing Cells

**Figure 2:**
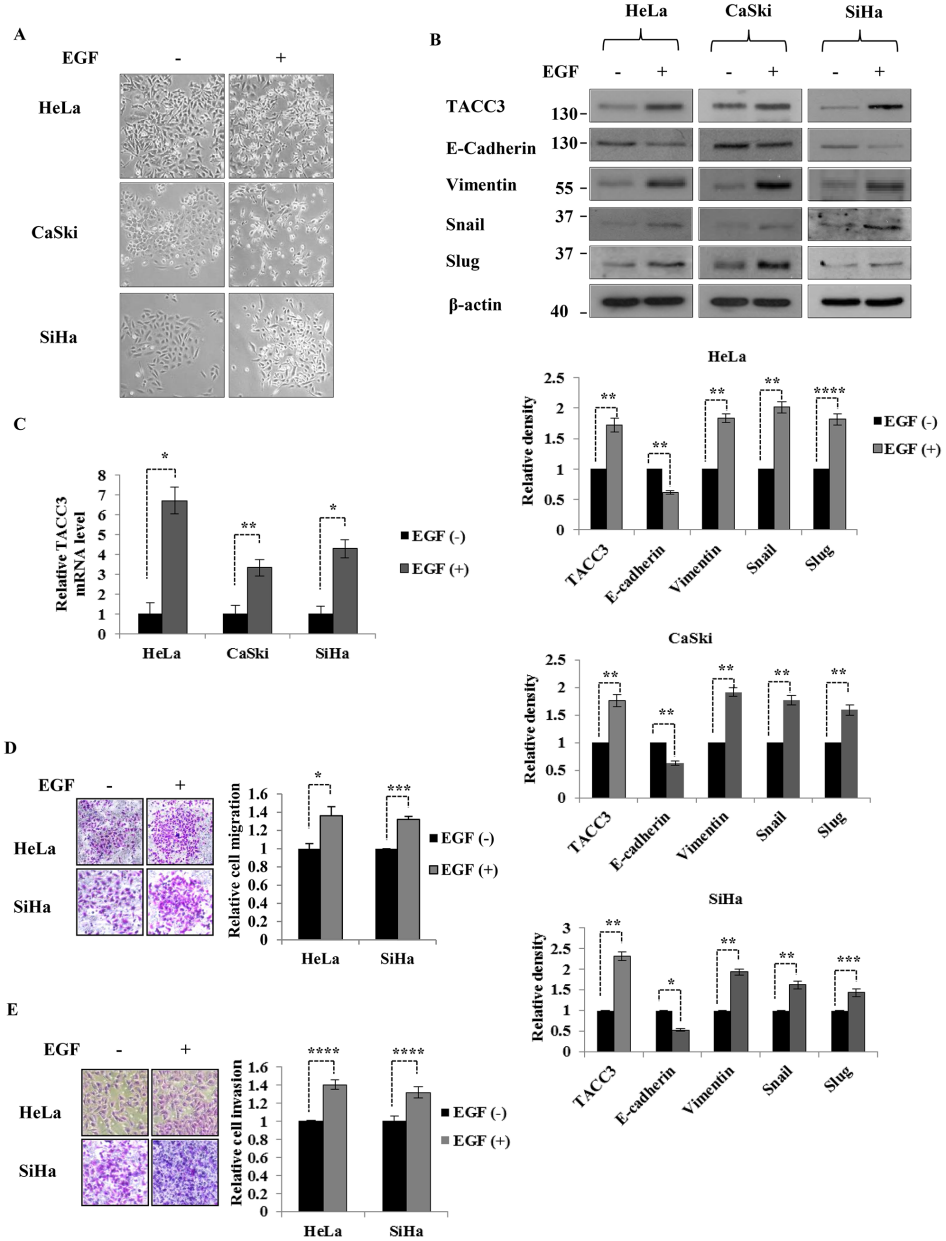
EGF stimulation induces the expression of TACC3. (**A**) Cervical cancer cells treated with EGF showed a morphological change associated with EMT. (**B and C**) Both protein (**B**) and mRNA (**C**) levels of TACC3 were up-regulated upon EGF stimulation, along with down-regulation of E-cadherin and up-regulation of Vimentin, Snail and Slug. β-actin was used as loading control. The intensity of bands was quantified using imageJ software and normalized to β-actin. The mRNA level of TACC3 was represented relative to β-actin transcripts. Data shown are means ± SD of at least three independent experiments. (**D and E**) HeLa and SiHa cells treated with or without EGF were subjected to transwell migration (**D**) and Matrigel invasion assays (**E**) (see Materials and Methods). Cells were incubated with or without 50 ng/ml of EGF for 24 h. Data shown are means ± SD of at least three independent experiments. *, *p*<0.05; **, *p*<0.01; ***, *p*<0.005; ****, *p*<0.001.

**Figure 3:**
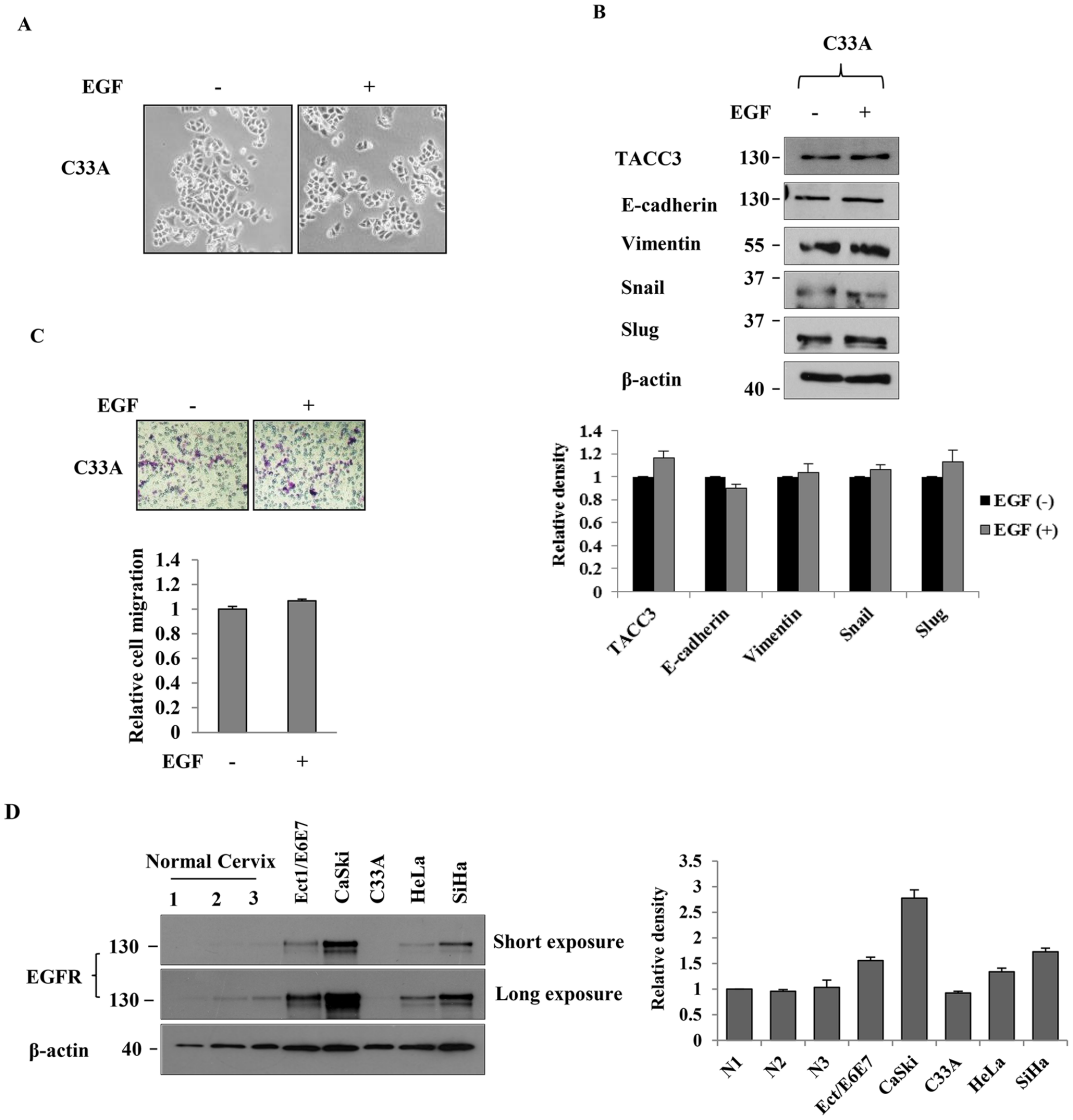
TACC3 is induced upon EGF stimulation in EGFR-expressing cells. C33A cells treated with EGF did not show significant changes in cell morphology (**A**), expression of TACC3 and EMT markers (**B**), or motility (**C**). Cells were incubated with or without 50 ng/ml of EGF for 24 h and then subjected to western blot and transwell migration assays. The intensity of bands was quantified using imageJ software and normalized to β-actin. Data shown are means ± SD of at least three independent experiments. (**D**) The expression of EGFR in Ect1/E6E7, CaSki, C33A, SiHa and HeLa cell lines was determined by western blot analysis. β-actin was used as a loading control. The intensity of bands was quantified using imageJ software and normalized to β-actin. Data shown are means ± SD of at least three independent experiments.

Our previous study indicated that overexpression of TACC3 induces EMT, accompanied by down-regulation of E-cadherin and up-regulation of Snail and Slug, whereas depletion of TACC3 reverses EMT [9]. In addition, the activation of Akt and ERK signaling pathways is essential for TACC3-mediated EMT [9]. Here, we sought to determine how TACC3 participates in EMT. Diverse growth factors, such as EGF, transforming growth factor β (TGF-β) and insulin-like growth factor 1 (IGF-1) have been shown to induce EMT and are significantly associated with the invasiveness, metastasis and recurrence of cervical cancer [15], [68]–[70]. EGF has been shown to induce EMT via up-regulation of Snail in cervical cancer cells [15] and activate Akt and ERK signaling pathways [71], [72]. Based upon these studies, we hypothesized that TACC3 may be involved in EGF-mediated EMT. To test our hypothesis, we treated HeLa, CaSki and SiHa cells with 50 ng/ml of EGF for 24 h and then examined the morphological changes of cells and the expression of the EMT markers. As shown in Figure 2A, cells treated with EGF displayed morphological and phenotypical features of EMT. Intriguingly, both protein (Figure 2B) and mRNA (Figure 2C) levels of TACC3 were significantly increased upon EGF stimulation, accompanied by down-regulation of epithelial marker E-cadherin and up-regulation of mesenchymal marker Vimentin as well as EMT inducers Snail and Slug in HeLa, CaSki and SiHa cells (Figure 2B). Time course experiments were also performed to verify the induction of TACC3 upon EGF stimulation. EGF induced a sustained expression of TACC3 in CaSki and SiHa cells up to 48 h, while a temporary increase in TACC3 expression was found in HeLa cells (Figure S2). We also found that EGF promoted migratory and invasive capabilities of HeLa and SiHa cells (Figure 2D and 2E), consistent with other studies [15], [73]. We did not see any significant changes in cell morphology (Figure 3A), the expression of TACC3 and other EMT markers (Figure 3B), or motility (Figure 3C) in EGF-treated C33A cells. This is most likely because C33A expresses very low or undetectable levels of EGFR (Figure 3D). These results suggest that EGFR is required for EGF-mediated induction of TACC3 and subsequent EMT.

### Activation of EGFR is Required for EGF-mediated Induction of TACC3

**Figure 4:**
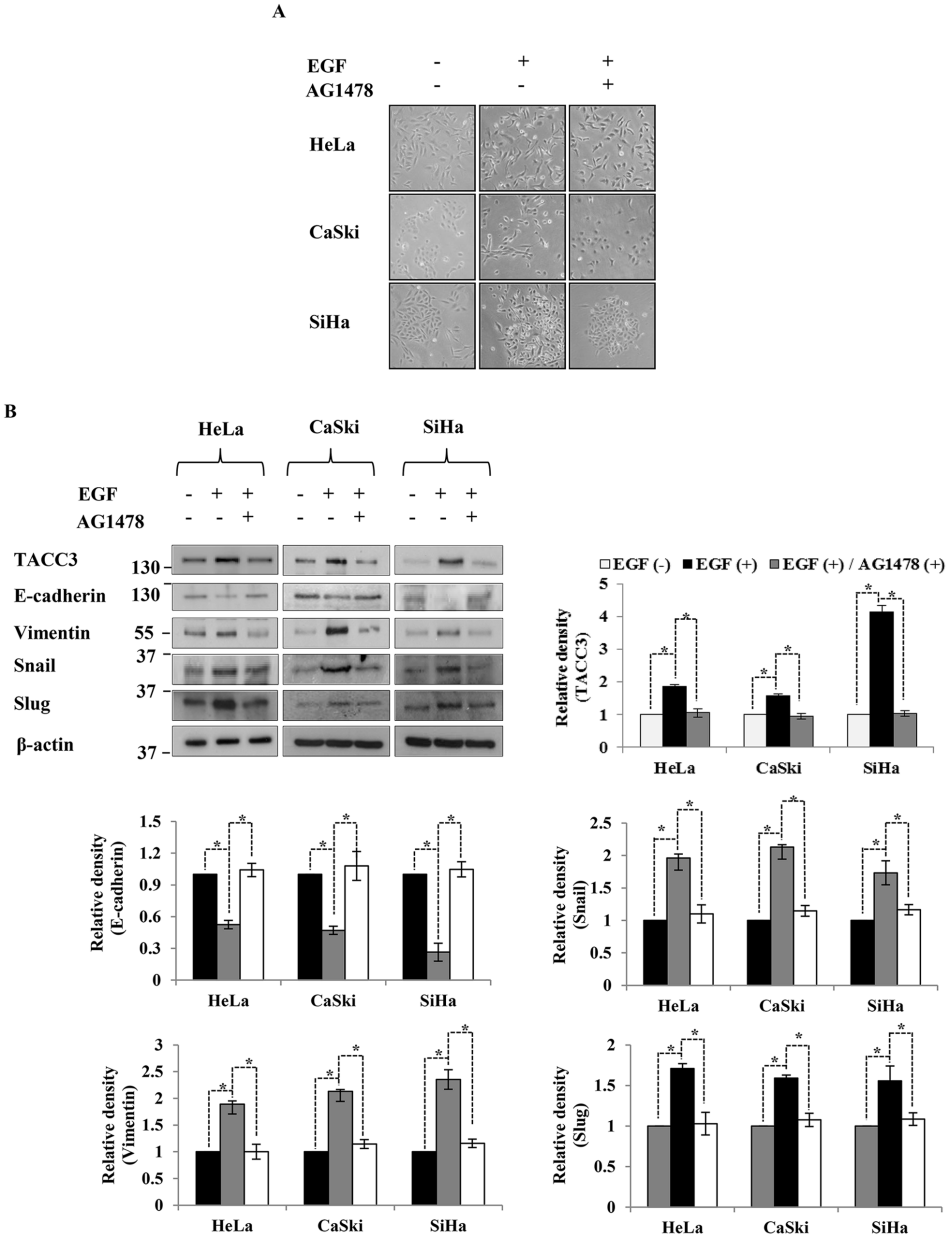
EGF-mediated TACC3 induction is dependent on EGFR activation. The inhibition of tyrosine kinase activity of EGFR abolished a morphological change associated with EMT (**A**) and EGF-mediated TACC3 induction (**B**). Cells were treated with EGF or EGF+AG1478 for 24 h and then subjected to western blot analysis. β-actin was used as a loading control. The intensity of bands was quantified using imageJ software and normalized to β-actin. Data shown are means ± SD of at least three independent experiments. *, *p*<0.001.

As our data indicates that EGF treatment can increase the expression of TACC3 in EGFR-expressing cells, we questioned whether EGF-mediated induction of TACC3 is dependent on the tyrosine kinase activity of EGFR. AG1478 is a tyrosine kinase inhibitor of EGFR, which blocks EGFR-mediated signaling events [74], [75]. Treatment with 5 µM of AG1478 abolished EGF-induced morphological changes (Figure 4A) and TACC3 induction (Figure 4B), and as a consequence, EGF-mediated EMT was inhibited (Figure 4B). This data suggests that activation of EGFR is required for EGF-mediated TACC3 induction.

### TACC3 is Required for EGF-mediated EMT Process

**Figure 5:**
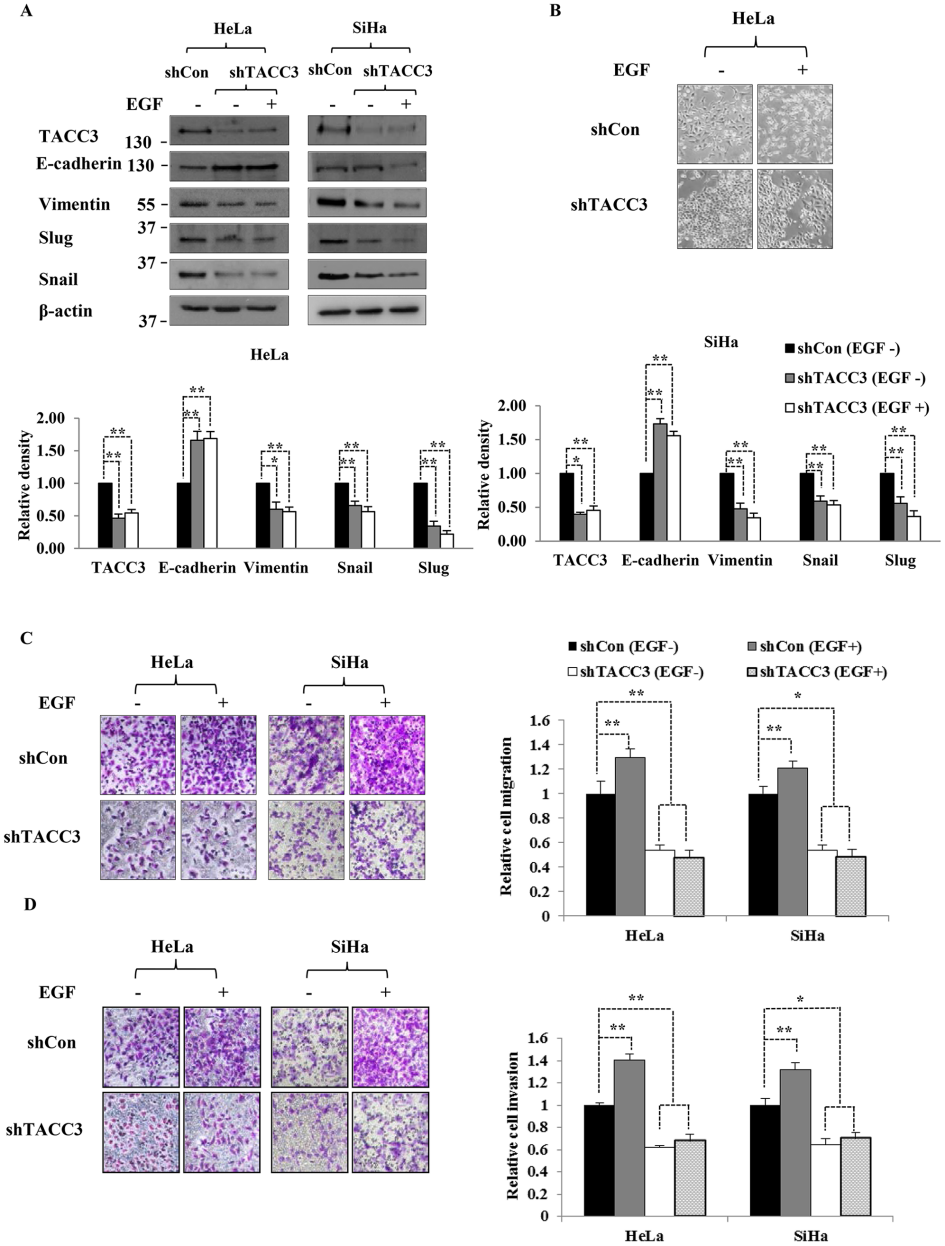
TACC3 is required for EGF-mediated EMT. In the absence of TACC3, EGF was not able to regulate EMT markers (**A**), alter cell morphology (**B**), or enhance cell migration and invasion capabilities (**C and D**). Cells were transiently transfected with shRNAs against control (scrambled, *shCon*) or TACC3 (*shTACC3*) and treated with or without EGF for 24 h. Cells were then subjected to western blot, transwell migration and Matrigel invasion assays. The intensity of bands was quantified using imageJ software and normalized to β-actin. Data shown are means ± SD of at least three independent experiments. *, *p<*0.01; **, p<0.001.

Since both TACC3 and EGF can promote EMT through the activation of PI3K/Akt and ERK signaling pathways [9], [71], [72], and TACC3 can be up-regulated upon EGF stimulation (Figure 2B, 2C and S2), we questioned whether TACC3 plays an important role in EGF-mediated EMT. To address this question, we depleted TACC3 using a lentiviral vector delivering shRNA specific to TACC3 in HeLa and SiHa cells and treated with or without EGF. As we previously reported [9], depletion of TACC3 led to the up-regulation of E-cadherin and down-regulation of Vimentin, Snail and Slug in HeLa and SiHa cells compared to control cells (Figure 5A). Intriguingly, EGF treatment on TACC3-depleted cells was not able to revert the expression of EMT markers (Figure 5A), cell morphology (Figure 5B), cell migration (Figure 5C) or invasion capacity (Figure 5D). These findings suggest that TACC3 may play a key role in EGF-mediated EMT.

### A Positive Correlation Exists between TACC3 and Snail Expression in Cervical Cancer

**Figure 6:**
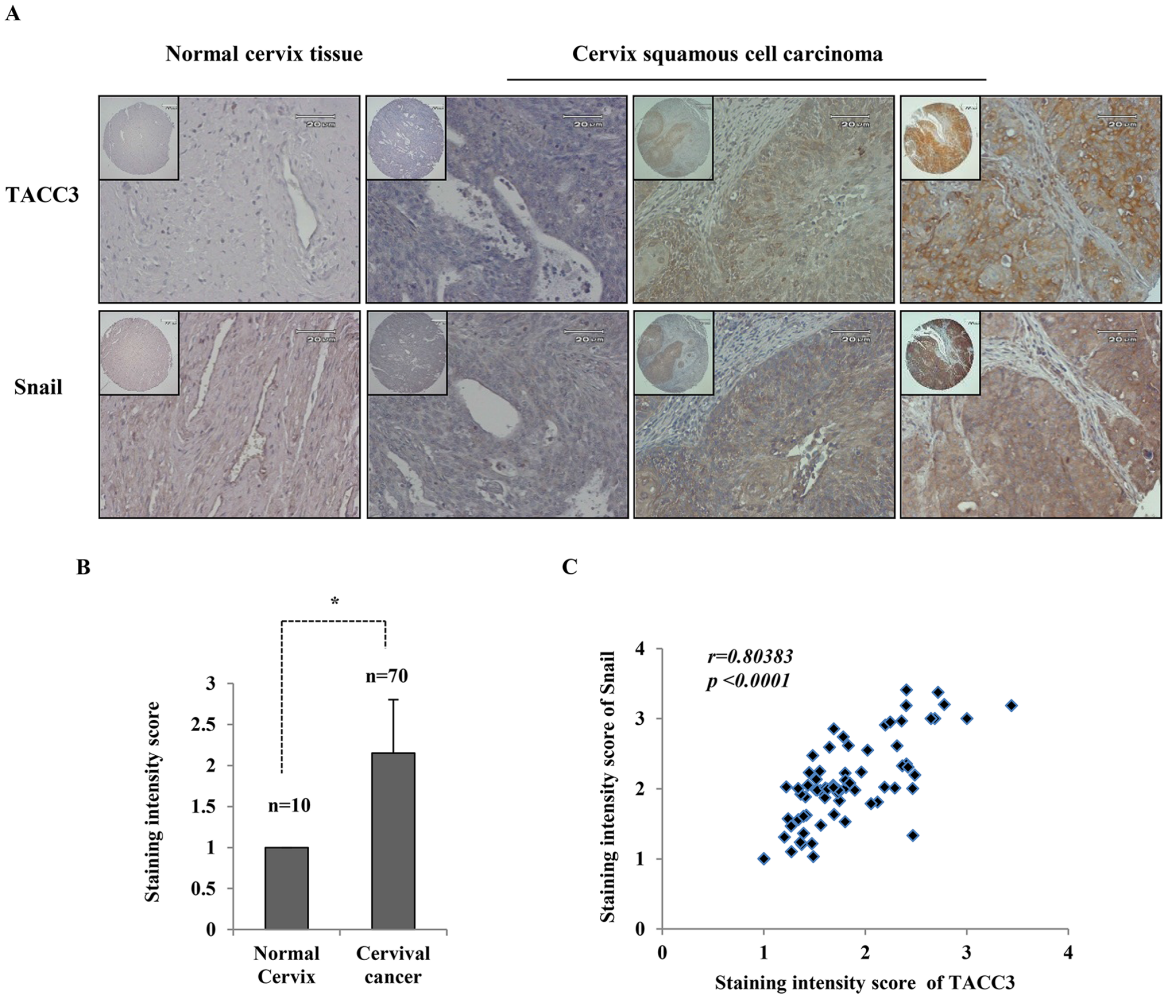
A correlation between TACC3 and Snail expression in cervical cancer tissue microarray. (**A**) Representative immunohistochemical staining of TACC3 and Snail on cervical cancer tissue microarray. Quantitative analysis of cervical cancer tissue microarrays showed that (**B**) the expression of Snail is higher in cervical cancer than in normal cervix. Data shown are means ± SD of at least three independent experiments. *, *p*<0.001 (**C**) Snail expression correlates with TACC3 expression (r = 0.80383, *p<0.0001*).

Snail has been known to be induced by EGF, thus triggering EGF-mediated EMT [15], [76]. We found that TACC3 was also induced upon EGF stimulation (Figure 2B and 2C) and positively regulates the expression of Snail [9]. Therefore, we questioned whether there is any correlation between TACC3 and Snail expression in cervical cancer. Importantly, we found that the expression of Snail was elevated in cervical cancer (Figure 6A and 6B) and correlated with TACC3 expression (Figure 6C). However, TACC3 expression did not correlate with the expression of Slug, another member of the Snail family (data not shown).

## Discussion

It has been suspected that deregulation (both up- and down-regulation) of TACC3 may be associated with the development of various types of human cancer [24], [77], [78]. So far, whether TACC3 acts as a tumor suppressor or an oncogene has not been clearly defined due to the discrepancies among studies [24], [79]. Alternatively, TACC3 may have different functions depending on the type of cell or organ. In this study, we aimed to investigate the functional significance of TACC3 in cervical cancer. Our cervical cancer tissue microarray analysis revealed that the expression of TACC3 protein is up-regulated in cervical squamous cell carcinoma, the most common type of cervical cancer (approximately 80–90%) [80]. This is consistent with data obtained from Oncomine [67] and suggests its potential role as an oncogene in cervical cancer. Our study also suggests that the expression of TACC3 may not be regulated by HPV E6/E7 oncogenes, however, at the moment, we cannot rule out the possibility that TACC3 expression is regulated by HPV oncogenes or correlated with the types of HPV.

EGF triggers a cascade of signaling events through interaction with its receptor, EGFR [81], and has been proven to be a potent inducer of EMT in cervical cancer [15]. EGF/EGFR signaling events are associated with accelerated tumor progression of cervical cancer [15], [19], [82]. In this study, we found that TACC3 was up-regulated upon EGF stimulation, and depletion of TACC3 abolished EGF-mediated EMT process in cervical cancer cells. Moreover, induction of TACC3 by EGF was successfully inhibited by the EGFR inhibitor AG1478, indicating that EGF-mediated TACC3 induction and subsequent EMT are dependent on EGFR activation. Interestingly, we found that in C33A cells, TACC3 expression was much higher than in CaSki or SiHa cells, despite the absence of EGFR expression. Although our study suggests that EGFR activation may be one of the mechanisms responsible for the regulation of the expression of TACC3, upstream signaling which regulates the expression of TACC3 has not yet been well defined. There has so far been only one published study demonstrating decreased TACC3 protein stability in a Cdh1-dependent manner [83]. TACC3 interacts with an activator of the E3 ubiquitin ligase anaphase-promoting complex/cyclosome (APC/C), Cdh1, and its interaction reduces TACC3 protein stability through Cdh1-mediated ubiquitination and degradation during cell cycle progression. One possibility we consider is that C33A cells may express lower levels of Cdh1 than other cells lines, thus maintaining high levels of TACC3. Interestingly, unlike SiHa, CaSki and HeLa cell lines, C33A cells do not harbor HPV genomes but contain mutated p53 [84] and pRb [85]. In addition, various growth factor receptors expressed in C33A are somewhat different from SiHa, CaSki and HeLa cells [86], [87]. Therefore, it is possible that TACC3 expression may be regulated by different signaling pathways depending on cell type.

There is a growing body of evidence demonstrating the association of TACC3 with EGFR signaling pathways. TACC3 has been identified as an interacting partner of the signal transducer and activator of transcription 5 (STAT5) [39], and its expression has been shown to be correlated with Aurora A expression in certain types of cancers [40], [63]. Intriguingly, EGFR also associates and cooperates with STAT5 to target and increase the expression of Aurora A, and its expression is found to be correlated with Aurora A expression in breast and colorectal cancers [81]. Therefore, it is reasonable to think that TACC3 may form a complex with EGFR directly or indirectly through its interaction with STAT5, and in that way be involved in EGFR signaling pathways. TACC3 was also discovered as an aryl hydrocarbon receptor (AhR) nuclear translocator (ARNT)-interacting protein [88]. ARNT belongs to the basic helix-loop-helix (bHLH)-Per-ARNT-Sim (PAS) family that dimerizes with AhR and hypoxia-inducible factor α (HIF α) upon environmental stress [89], [90]. As a transcription cofactor, TACC3 is able to regulate the transcriptional activation of HIF through direct interaction with ARNT [91]. A recent study has shown that ARNT plays an important role in epidermal differentiation through regulation of the EGFR-ERK signaling pathways [92]. Thus, it is tempting to speculate a potential involvement of TACC3 in the network of ARNT-EGFR-ERK to regulate keratinocyte differentiation.

EGFR is expressed at moderate to high levels in cervical cancer, and its expression is associated with clinical stage and poor prognosis [93], [94]. Mutations in *EGFR* have been shown to be rare in high-grade invasive cervical cancer [95]. So far, only a few small-scale clinical trials have been tested to evaluate the efficacy of EGFR inhibitors for the treatment of cervical cancer. Unfortunately, EGFR inhibitor monotherapy was not efficient in patients with recurring locoregionally advanced or metastatic cervical cancer [94], [96]. The combinations of EGFR inhibitors with chemotherapy or chemoradiotherapy are currently under investigation.

Since TACC3 is overexpressed in cervical and other cancers and appears to be a key player in EGF/EGFR-driven EMT process, it is possible that depletion of TACC3 may be a good approach to treat cancers that are driven by EGF/EGFR signaling pathways or resistant to anti-EGFR therapy. Although mutations in *EGFR* in high-grade invasive cervical cancer are rare, it would be important to study an association between TACC3 expression and *EGFR* mutations. Overall, our findings suggest that TACC3 plays an important role in EGF-mediated EMT and may serve as an attractive therapeutic target for human cancers driven by EGF/EGFR signaling pathways.

## Supporting Information

Figure S1 **The expression of TACC3 in cervical cancer with respect to stage of the disease and histological grading.** The expression of TACC3 with different disease stages and tumor grade was presented. *, *p*<0.05; **, *p*<0.01; ***, *p*<0.001
(TIF)

Figure S2 **EGF stimulation induces endogenous TACC3 expression.** TACC3 was induced by EGF treatment. Cells were incubated with or without 50 ng/ml and then collected at the indicated time points for western blot analysis. β-actin was used as a loading control.
(TIF)

## References

[1] Turley EA, Veiseh M, Radisky DC, Bissell MJ (2008) Mechanisms of disease: epithelial-mesenchymal transition–does cellular plasticity fuel neoplastic progression? Nat Clin Pract Oncol 5: 280–290. 18349857 10.1038/ncponc1089 PMC2846172

[2] Kalluri R, Weinberg RA (2009) The basics of epithelial-mesenchymal transition. J Clin Invest 119: 1420–1428. 19487818 10.1172/JCI39104 PMC2689101

[3] Huber MA, Kraut N, Beug H (2005) Molecular requirements for epithelial-mesenchymal transition during tumor progression. Curr Opin Cell Biol 17: 548–558. 16098727 10.1016/j.ceb.2005.08.001

[4] Thiery JP, Acloque H, Huang RY, Nieto MA (2009) Epithelial-mesenchymal transitions in development and disease. Cell 139: 871–890. 19945376 10.1016/j.cell.2009.11.007

[5] Tanos B, Rodriguez-Boulan E (2008) The epithelial polarity program: machineries involved and their hijacking by cancer. Oncogene 27: 6939–6957. 19029936 10.1038/onc.2008.345

[6] Cheng JC, Auersperg N, Leung PC (2012) EGF-induced EMT and invasiveness in serous borderline ovarian tumor cells: a possible step in the transition to low-grade serous carcinoma cells? PLoS One 7: e34071. 22479527 10.1371/journal.pone.0034071 PMC3316602

[7] Kalluri R (2009) EMT: when epithelial cells decide to become mesenchymal-like cells. J Clin Invest 119: 1417–1419. 19487817 10.1172/JCI39675 PMC2689122

[8] Kang Y, Massague J (2004) Epithelial-mesenchymal transitions: twist in development and metastasis. Cell 118: 277–279. 15294153 10.1016/j.cell.2004.07.011

[9] Ha GH, Park JS, Breuer EK (2013) TACC3 promotes epithelial-mesenchymal transition (EMT) through the activation of PI3K/Akt and ERK signaling pathways. Cancer Lett 332: 63–73. 23348690 10.1016/j.canlet.2013.01.013

[10] Batlle E, Sancho E, Franci C, Dominguez D, Monfar M, et al (2000) The transcription factor snail is a repressor of E-cadherin gene expression in epithelial tumour cells. Nat Cell Biol 2: 84–89. 10655587 10.1038/35000034

[11] Hajra KM, Chen DY, Fearon ER (2002) The SLUG zinc-finger protein represses E-cadherin in breast cancer. Cancer Res 62: 1613–1618. 11912130

[12] Yang J, Mani SA, Donaher JL, Ramaswamy S, Itzykson RA, et al (2004) Twist, a master regulator of morphogenesis, plays an essential role in tumor metastasis. Cell 117: 927–939. 15210113 10.1016/j.cell.2004.06.006

[13] Eger A, Aigner K, Sonderegger S, Dampier B, Oehler S, et al (2005) DeltaEF1 is a transcriptional repressor of E-cadherin and regulates epithelial plasticity in breast cancer cells. Oncogene 24: 2375–2385. 15674322 10.1038/sj.onc.1208429

[14] Byles V, Zhu L, Lovaas JD, Chmilewski LK, Wang J, et al (2012) SIRT1 induces EMT by cooperating with EMT transcription factors and enhances prostate cancer cell migration and metastasis. Oncogene 31: 4619–4629. 22249256 10.1038/onc.2011.612 PMC4157820

[15] Lee MY, Chou CY, Tang MJ, Shen MR (2008) Epithelial-mesenchymal transition in cervical cancer: correlation with tumor progression, epidermal growth factor receptor overexpression, and snail up-regulation. Clin Cancer Res 14: 4743–4750. 18676743 10.1158/1078-0432.CCR-08-0234

[16] Ali SF, Ayub S, Manzoor NF, Azim S, Afif M, et al (2010) Knowledge and awareness about cervical cancer and its prevention amongst interns and nursing staff in Tertiary Care Hospitals in Karachi, Pakistan. PLoS One 5: e11059. 20548787 10.1371/journal.pone.0011059 PMC2883573

[17] zur Hausen H (1991) Human papillomaviruses in the pathogenesis of anogenital cancer. Virology 184: 9–13. 1651607 10.1016/0042-6822(91)90816-t

[18] Burd EM (2003) Human papillomavirus and cervical cancer. Clin Microbiol Rev 16: 1–17. 12525422 10.1128/CMR.16.1.1-17.2003 PMC145302

[19] Myong NH (2012) Loss of E-cadherin and Acquisition of Vimentin in Epithelial-Mesenchymal Transition are Noble Indicators of Uterine Cervix Cancer Progression. Korean J Pathol 46: 341–348. 23110026 10.4132/KoreanJPathol.2012.46.4.341 PMC3479821

[20] Lee MY, Shen MR (2012) Epithelial-mesenchymal transition in cervical carcinoma. Am J Transl Res 4: 1–13. 22347518 PMC3276374

[21] Schrevel M, Gorter A, Kolkman-Uljee SM, Trimbos JB, Fleuren GJ, et al (2011) Molecular mechanisms of epidermal growth factor receptor overexpression in patients with cervical cancer. Mod Pathol 24: 720–728. 21252859 10.1038/modpathol.2010.239

[22] Gergely F, Karlsson C, Still I, Cowell J, Kilmartin J, et al (2000) The TACC domain identifies a family of centrosomal proteins that can interact with microtubules. Proc Natl Acad Sci U S A 97: 14352–14357. 11121038 10.1073/pnas.97.26.14352 PMC18922

[23] O'Brien LL, Albee AJ, Liu L, Tao W, Dobrzyn P, et al (2005) The Xenopus TACC homologue, maskin, functions in mitotic spindle assembly. Mol Biol Cell 16: 2836–2847. 15788567 10.1091/mbc.E04-10-0926 PMC1142428

[24] Ha GH, Kim JL, Breuer EK (2013) Transforming acidic coiled-coil proteins (TACCs) in human cancer. Cancer Lett 336: 24–33. 23624299 10.1016/j.canlet.2013.04.022

[25] Lee MJ, Gergely F, Jeffers K, Peak-Chew SY, Raff JW (2001) Msps/XMAP215 interacts with the centrosomal protein D-TACC to regulate microtubule behaviour. Nat Cell Biol 3: 643–649. 11433296 10.1038/35083033

[26] Sadek CM, Pelto-Huikko M, Tujague M, Steffensen KR, Wennerholm M, et al (2003) TACC3 expression is tightly regulated during early differentiation. Gene Expr Patterns 3: 203–211. 12711550 10.1016/s1567-133x(02)00066-2

[27] Schneider L, Essmann F, Kletke A, Rio P, Hanenberg H, et al (2007) The transforming acidic coiled coil 3 protein is essential for spindle-dependent chromosome alignment and mitotic survival. J Biol Chem 282: 29273–29283. 17675670 10.1074/jbc.M704151200

[28] Gergely F, Draviam VM, Raff JW (2003) The ch-TOG/XMAP215 protein is essential for spindle pole organization in human somatic cells. Genes Dev 17: 336–341. 12569123 10.1101/gad.245603 PMC195983

[29] Yao R, Natsume Y, Noda T (2007) TACC3 is required for the proper mitosis of sclerotome mesenchymal cells during formation of the axial skeleton. Cancer Sci 98: 555–562. 17359303 10.1111/j.1349-7006.2007.00433.x PMC11158658

[30] Still IH, Hamilton M, Vince P, Wolfman A, Cowell JK (1999) Cloning of TACC1, an embryonically expressed, potentially transforming coiled coil containing gene, from the 8p11 breast cancer amplicon. Oncogene 18: 4032–4038. 10435627 10.1038/sj.onc.1202801

[31] Lauffart B, Gangisetty O, Still IH (2003) Molecular cloning, genomic structure and interactions of the putative breast tumor suppressor TACC2. Genomics 81: 192–201. 12620397 10.1016/s0888-7543(02)00039-3

[32] Still IH, Vince P, Cowell JK (1999) The third member of the transforming acidic coiled coil-containing gene family, TACC3, maps in 4p16, close to translocation breakpoints in multiple myeloma, and is upregulated in various cancer cell lines. Genomics 58: 165–170. 10366448 10.1006/geno.1999.5829

[33] Albee AJ, Wiese C (2008) Xenopus TACC3/maskin is not required for microtubule stability but is required for anchoring microtubules at the centrosome. Mol Biol Cell 19: 3347–3356. 18508920 10.1091/mbc.E07-11-1204 PMC2488304

[34] Kinoshita K, Noetzel TL, Pelletier L, Mechtler K, Drechsel DN, et al (2005) Aurora A phosphorylation of TACC3/maskin is required for centrosome-dependent microtubule assembly in mitosis. J Cell Biol 170: 1047–1055. 16172205 10.1083/jcb.200503023 PMC2171544

[35] Gomez-Baldo L, Schmidt S, Maxwell CA, Bonifaci N, Gabaldon T, et al (2010) TACC3-TSC2 maintains nuclear envelope structure and controls cell division. Cell Cycle 9: 1143–1155. 20237422 10.4161/cc.9.6.11018

[36] Bargo S, Raafat A, McCurdy D, Amirjazil I, Shu Y, et al (2010) Transforming acidic coiled-coil protein-3 (Tacc3) acts as a negative regulator of Notch signaling through binding to CDC10/Ankyrin repeats. Biochem Biophys Res Commun 400: 606–612. 20804727 10.1016/j.bbrc.2010.08.111 PMC2964058

[37] Angrisano T, Lembo F, Pero R, Natale F, Fusco A, et al (2006) TACC3 mediates the association of MBD2 with histone acetyltransferases and relieves transcriptional repression of methylated promoters. Nucleic Acids Res 34: 364–372. 16410616 10.1093/nar/gkj400 PMC1331987

[38] Garriga-Canut M, Orkin SH (2004) Transforming acidic coiled-coil protein 3 (TACC3) controls friend of GATA-1 (FOG-1) subcellular localization and regulates the association between GATA-1 and FOG-1 during hematopoiesis. J Biol Chem 279: 23597–23605. 15037632 10.1074/jbc.M313987200

[39] Piekorz RP, Hoffmeyer A, Duntsch CD, McKay C, Nakajima H, et al (2002) The centrosomal protein TACC3 is essential for hematopoietic stem cell function and genetically interfaces with p53-regulated apoptosis. EMBO J 21: 653–664. 11847113 10.1093/emboj/21.4.653 PMC125348

[40] Duncan CG, Killela PJ, Payne CA, Lampson B, Chen WC, et al (2010) Integrated genomic analyses identify ERRFI1 and TACC3 as glioblastoma-targeted genes. Oncotarget 1: 265–277. 21113414 10.18632/oncotarget.137 PMC2992381

[41] Kiemeney LA, Sulem P, Besenbacher S, Vermeulen SH, Sigurdsson A, et al (2010) A sequence variant at 4p16.3 confers susceptibility to urinary bladder cancer. Nat Genet 42: 415–419. 20348956 10.1038/ng.558 PMC2923020

[42] Malgeri U, Baldini L, Perfetti V, Fabris S, Vignarelli MC, et al (2000) Detection of t(4;14)(p16.3;q32) chromosomal translocation in multiple myeloma by reverse transcription-polymerase chain reaction analysis of IGH-MMSET fusion transcripts. Cancer Res 60: 4058–4061. 10945609

[43] Wang M, Chu H, Yan F, Qin C, Li P, et al (2011) Chromosome 4p16.3 variant modify bladder cancer risk in a Chinese population. Carcinogenesis 32: 872–875. 21459758 10.1093/carcin/bgr060 PMC3314283

[44] Gusella JF, Wexler NS, Conneally PM, Naylor SL, Anderson MA, et al (1983) A polymorphic DNA marker genetically linked to Huntington's disease. Nature 306: 234–238. 6316146 10.1038/306234a0

[45] Finelli P, Fabris S, Zagano S, Baldini L, Intini D, et al (1999) Detection of t(4;14)(p16.3;q32) chromosomal translocation in multiple myeloma by double-color fluorescent in situ hybridization. Blood 94: 724–732. 10397739

[46] Castro P, Creighton CJ, Ozen M, Berel D, Mims MP, et al (2009) Genomic profiling of prostate cancers from African American men. Neoplasia 11: 305–312. 19242612 10.1593/neo.81530 PMC2647733

[47] Shivapurkar N, Sood S, Wistuba, II, Virmani AK, Maitra A, et al (1999) Multiple regions of chromosome 4 demonstrating allelic losses in breast carcinomas. Cancer Res 59: 3576–3580. 10446964

[48] Shivapurkar N, Maitra A, Milchgrub S, Gazdar AF (2001) Deletions of chromosome 4 occur early during the pathogenesis of colorectal carcinoma. Hum Pathol 32: 169–177. 11230704 10.1053/hupa.2001.21560

[49] Singh D, Chan JM, Zoppoli P, Niola F, Sullivan R, et al (2012) Transforming Fusions of FGFR and TACC Genes in Human Glioblastoma. Science 337 1231–1235. 22837387 10.1126/science.1220834 PMC3677224

[50] Williams SV, Hurst CD, Knowles MA (2012) Oncogenic FGFR3 gene fusions in bladder cancer. Hum Mol Genet 22 795–803. 23175443 10.1093/hmg/dds486 PMC3554204

[51] Parsons DW, Jones S, Zhang X, Lin JC, Leary RJ, et al (2008) An integrated genomic analysis of human glioblastoma multiforme. Science 321: 1807–1812. 18772396 10.1126/science.1164382 PMC2820389

[52] Lauffart B, Vaughan MM, Eddy R, Chervinsky D, DiCioccio RA, et al (2005) Aberrations of TACC1 and TACC3 are associated with ovarian cancer. BMC Womens Health 5: 8. 15918899 10.1186/1472-6874-5-8 PMC1175095

[53] Jung CK, Jung JH, Park GS, Lee A, Kang CS, et al (2006) Expression of transforming acidic coiled-coil containing protein 3 is a novel independent prognostic marker in non-small cell lung cancer. Pathol Int 56: 503–509. 16930330 10.1111/j.1440-1827.2006.01998.x

[54] Stewart JP, Thompson A, Santra M, Barlogie B, Lappin TR, et al (2004) Correlation of TACC3, FGFR3, MMSET and p21 expression with the t(4;14)(p16.3;q32) in multiple myeloma. Br J Haematol 126: 72–76. 15198734 10.1111/j.1365-2141.2004.04996.x

[55] Kang H, Wilson CS, Harvey RC, Chen IM, Murphy MH, et al (2012) Gene expression profiles predictive of outcome and age in infant acute lymphoblastic leukemia: a Children's Oncology Group study. Blood 119: 1872–1881. 22210879 10.1182/blood-2011-10-382861 PMC3293641

[56] Chakraborty J, Okonta H, Bagalb H, Lee SJ, Fink B, et al (2008) Retroviral gene insertion in breast milk mediated lymphomagenesis. Virology 377: 100–109. 18501945 10.1016/j.virol.2008.04.008

[57] Ma XJ, Salunga R, Tuggle JT, Gaudet J, Enright E, et al (2003) Gene expression profiles of human breast cancer progression. Proc Natl Acad Sci U S A 100: 5974–5979. 12714683 10.1073/pnas.0931261100 PMC156311

[58] Peters DG, Kudla DM, Deloia JA, Chu TJ, Fairfull L, et al (2005) Comparative gene expression analysis of ovarian carcinoma and normal ovarian epithelium by serial analysis of gene expression. Cancer Epidemiol Biomarkers Prev 14: 1717–1723. 16030107 10.1158/1055-9965.EPI-04-0704

[59] L'Esperance S, Popa I, Bachvarova M, Plante M, Patten N, et al (2006) Gene expression profiling of paired ovarian tumors obtained prior to and following adjuvant chemotherapy: molecular signatures of chemoresistant tumors. Int J Oncol 29: 5–24. 16773180

[60] Yim EK, Tong SY, Ho EM, Bae JH, Um SJ, et al (2009) Anticancer effects on TACC3 by treatment of paclitaxel in HPV-18 positive cervical carcinoma cells. Oncol Rep 21: 549–557. 19148534

[61] Morton JP, Timpson P, Karim SA, Ridgway RA, Athineos D, et al (2010) Mutant p53 drives metastasis and overcomes growth arrest/senescence in pancreatic cancer. Proc Natl Acad Sci U S A 107: 246–251. 20018721 10.1073/pnas.0908428107 PMC2806749

[62] Livak KJ, Schmittgen TD (2001) Analysis of relative gene expression data using real-time quantitative PCR and the 2(−Delta Delta C(T)) Method. Methods 25: 402–408. 11846609 10.1006/meth.2001.1262

[63] Ulisse S, Baldini E, Toller M, Delcros JG, Gueho A, et al (2007) Transforming acidic coiled-coil 3 and Aurora-A interact in human thyrocytes and their expression is deregulated in thyroid cancer tissues. Endocr Relat Cancer 14: 827–837. 17914111 10.1677/ERC-07-0053 PMC2216418

[64] Lee HJ, Hwang HI, Jang YJ (2010) Mitotic DNA damage response: Polo-like kinase-1 is dephosphorylated through ATM-Chk1 pathway. Cell Cycle 9: 2389–2398. 20581453 10.4161/cc.9.12.11904

[65] Rhodes DR, Kalyana-Sundaram S, Mahavisno V, Varambally R, Yu J, et al (2007) Oncomine 3.0: genes, pathways, and networks in a collection of 18,000 cancer gene expression profiles. Neoplasia 9: 166–180. 17356713 10.1593/neo.07112 PMC1813932

[66] Scotto L, Narayan G, Nandula SV, Arias-Pulido H, Subramaniyam S, et al (2008) Identification of copy number gain and overexpressed genes on chromosome arm 20q by an integrative genomic approach in cervical cancer: potential role in progression. Genes Chromosomes Cancer 47: 755–765. 18506748 10.1002/gcc.20577

[67] Zhai Y, Kuick R, Nan B, Ota I, Weiss SJ, et al (2007) Gene expression analysis of preinvasive and invasive cervical squamous cell carcinomas identifies HOXC10 as a key mediator of invasion. Cancer Res 67: 10163–10172. 17974957 10.1158/0008-5472.CAN-07-2056

[68] Schonrath K, Klein-Szanto AJ, Braunewell KH (2012) The putative tumor suppressor VILIP-1 counteracts epidermal growth factor-induced epidermal-mesenchymal transition in squamous carcinoma cells. PLoS One 7: e33116. 22479362 10.1371/journal.pone.0033116 PMC3316558

[69] Wendt MK, Smith JA, Schiemann WP (2010) Transforming growth factor-beta-induced epithelial-mesenchymal transition facilitates epidermal growth factor-dependent breast cancer progression. Oncogene 29: 6485–6498. 20802523 10.1038/onc.2010.377 PMC3076082

[70] Graham TR, Zhau HE, Odero-Marah VA, Osunkoya AO, Kimbro KS, et al (2008) Insulin-like growth factor-I-dependent up-regulation of ZEB1 drives epithelial-to-mesenchymal transition in human prostate cancer cells. Cancer Res 68: 2479–2488. 18381457 10.1158/0008-5472.CAN-07-2559

[71] Gan Y, Shi C, Inge L, Hibner M, Balducci J, et al (2010) Differential roles of ERK and Akt pathways in regulation of EGFR-mediated signaling and motility in prostate cancer cells. Oncogene 29: 4947–4958. 20562913 10.1038/onc.2010.240

[72] Ahmed N, Maines-Bandiera S, Quinn MA, Unger WG, Dedhar S, et al (2006) Molecular pathways regulating EGF-induced epithelio-mesenchymal transition in human ovarian surface epithelium. Am J Physiol Cell Physiol 290: C1532–1542. 16394028 10.1152/ajpcell.00478.2005

[73] Chiang Y, Chou CY, Hsu KF, Huang YF, Shen MR (2008) EGF upregulates Na+/H+ exchanger NHE1 by post-translational regulation that is important for cervical cancer cell invasiveness. J Cell Physiol 214: 810–819. 17894388 10.1002/jcp.21277

[74] Johns TG, Luwor RB, Murone C, Walker F, Weinstock J, et al (2003) Antitumor efficacy of cytotoxic drugs and the monoclonal antibody 806 is enhanced by the EGF receptor inhibitor AG1478. Proc Natl Acad Sci U S A 100: 15871–15876. 14676326 10.1073/pnas.2036503100 PMC307660

[75] Gan HK, Walker F, Burgess AW, Rigopoulos A, Scott AM, et al (2007) The epidermal growth factor receptor (EGFR) tyrosine kinase inhibitor AG1478 increases the formation of inactive untethered EGFR dimers. Implications for combination therapy with monoclonal antibody 806. J Biol Chem 282: 2840–2850. 17092939 10.1074/jbc.M605136200

[76] Barrallo-Gimeno A, Nieto MA (2005) The Snail genes as inducers of cell movement and survival: implications in development and cancer. Development 132: 3151–3161. 15983400 10.1242/dev.01907

[77] Line A, Slucka Z, Stengrevics A, Li G, Rees RC (2002) Altered splicing pattern of TACC1 mRNA in gastric cancer. Cancer Genet Cytogenet 139: 78–83. 12547166 10.1016/s0165-4608(02)00607-6

[78] Raff JW (2002) Centrosomes and cancer: lessons from a TACC. Trends Cell Biol 12: 222–225. 12062169 10.1016/s0962-8924(02)02268-7

[79] Guyot R, Vincent S, Bertin J, Samarut J, Ravel-Chapuis P (2010) The transforming acidic coiled coil (TACC1) protein modulates the transcriptional activity of the nuclear receptors TR and RAR. BMC Mol Biol 11: 3. 20078863 10.1186/1471-2199-11-3 PMC2822774

[80] International Collaboration of Epidemiological Studies of Cervical C (2007) Comparison of risk factors for invasive squamous cell carcinoma and adenocarcinoma of the cervix: collaborative reanalysis of individual data on 8,097 women with squamous cell carcinoma and 1,374 women with adenocarcinoma from 12 epidemiological studies. Int J Cancer 120: 885–891. 17131323 10.1002/ijc.22357

[81] Hung LY, Tseng JT, Lee YC, Xia W, Wang YN, et al (2008) Nuclear epidermal growth factor receptor (EGFR) interacts with signal transducer and activator of transcription 5 (STAT5) in activating Aurora-A gene expression. Nucleic Acids Res 36: 4337–4351. 18586824 10.1093/nar/gkn417 PMC2490761

[82] Shen L, Shui Y, Wang X, Sheng L, Yang Z, et al (2008) EGFR and HER2 expression in primary cervical cancers and corresponding lymph node metastases: implications for targeted radiotherapy. BMC Cancer 8: 232. 18700025 10.1186/1471-2407-8-232 PMC2519090

[83] Jeng JC, Lin YM, Lin CH, Shih HM (2009) Cdh1 controls the stability of TACC3. Cell Cycle 8: 3529–3536. 10.4161/cc.8.21.9935 19823035

[84] Hougardy BM, van der Zee AG, van den Heuvel FA, Timmer T, de Vries EG, et al (2005) Sensitivity to Fas-mediated apoptosis in high-risk HPV-positive human cervical cancer cells: relationship with Fas, caspase-8, and Bid. Gynecol Oncol 97: 353–364. 15863130 10.1016/j.ygyno.2005.01.036

[85] Scheffner M, Munger K, Byrne JC, Howley PM (1991) The state of the p53 and retinoblastoma genes in human cervical carcinoma cell lines. Proc Natl Acad Sci U S A 88: 5523–5527. 1648218 10.1073/pnas.88.13.5523 PMC51909

[86] Serrano ML, Sanchez-Gomez M, Bravo MM, Yakar S, LeRoith D (2008) Differential expression of IGF-I and insulin receptor isoforms in HPV positive and negative human cervical cancer cell lines. Horm Metab Res 40: 661–667. 18711691 10.1055/s-0028-1082080

[87] Shen MR, Hsu YM, Hsu KF, Chen YF, Tang MJ, et al (2006) Insulin-like growth factor 1 is a potent stimulator of cervical cancer cell invasiveness and proliferation that is modulated by alphavbeta3 integrin signaling. Carcinogenesis 27: 962–971. 16400188 10.1093/carcin/bgi336

[88] Sadek CM, Jalaguier S, Feeney EP, Aitola M, Damdimopoulos AE, et al (2000) Isolation and characterization of AINT: a novel ARNT interacting protein expressed during murine embryonic development. Mech Dev 97: 13–26. 11025203 10.1016/s0925-4773(00)00415-9

[89] Kewley RJ, Whitelaw ML, Chapman-Smith A (2004) The mammalian basic helix-loop-helix/PAS family of transcriptional regulators. Int J Biochem Cell Biol 36: 189–204. 14643885 10.1016/s1357-2725(03)00211-5

[90] Conte N, Charafe-Jauffret E, Delaval B, Adelaide J, Ginestier C, et al (2002) Carcinogenesis and translational controls: TACC1 is down-regulated in human cancers and associates with mRNA regulators. Oncogene 21: 5619–5630. 12165861 10.1038/sj.onc.1205658

[91] Partch CL, Gardner KH (2011) Coactivators necessary for transcriptional output of the hypoxia inducible factor, HIF, are directly recruited by ARNT PAS-B. Proc Natl Acad Sci U S A 108: 7739–7744. 21512126 10.1073/pnas.1101357108 PMC3093465

[92] Robertson ED, Weir L, Romanowska M, Leigh IM, Panteleyev AA (2012) ARNT controls the expression of epidermal differentiation genes through HDAC- and EGFR-dependent pathways. J Cell Sci 125: 3320–3332. 22505606 10.1242/jcs.095125

[93] Kersemaekers AM, Fleuren GJ, Kenter GG, Van den Broek LJ, Uljee SM, et al (1999) Oncogene alterations in carcinomas of the uterine cervix: overexpression of the epidermal growth factor receptor is associated with poor prognosis. Clin Cancer Res 5: 577–586. 10100709

[94] Goncalves A, Fabbro M, Lhomme C, Gladieff L, Extra JM, et al (2008) A phase II trial to evaluate gefitinib as second- or third-line treatment in patients with recurring locoregionally advanced or metastatic cervical cancer. Gynecol Oncol 108: 42–46. 17980406 10.1016/j.ygyno.2007.07.057

[95] Soonthornthum T, Arias-Pulido H, Joste N, Lomo L, Muller C, et al (2011) Epidermal growth factor receptor as a biomarker for cervical cancer. Ann Oncol 22: 2166–2178. 21325449 10.1093/annonc/mdq723

[96] Hertlein L, Lenhard M, Kirschenhofer A, Kahlert S, Mayr D, et al (2011) Cetuximab monotherapy in advanced cervical cancer: a retrospective study with five patients. Arch Gynecol Obstet 283: 109–113. 20180130 10.1007/s00404-010-1389-1

