# Correction: TACC3 Is Essential for EGF-Mediated EMT in Cervical Cancer

**DOI:** 10.1371/annotation/23c9bde1-5ced-4eb9-8a73-c53f3f2913d4

**Published:** 2013-09-20

**Authors:** Geun-Hyoung Ha, Jung-Lye Kim, Eun-Kyoung Breuer

The name of the third author was incorrectly represented in the Citation. The correct Citation is: Ha G-H, Kim J-L, Breuer E-K (2013) TACC3 Is Essential for EGF-Mediated EMT in Cervical Cancer. PLoS ONE 8(8): e70353. doi:10.1371/journal.pone.0070353.

