# Supplementary figures and images for: TACC3 Is Essential for EGF-Mediated EMT in Cervical Cancer
Source: PLoS One. 2013 Aug 1;8(8):e70353. doi: 10.1371/journal.pone.0070353 (PMC3731346; doi:10.1371/journal.pone.0070353)

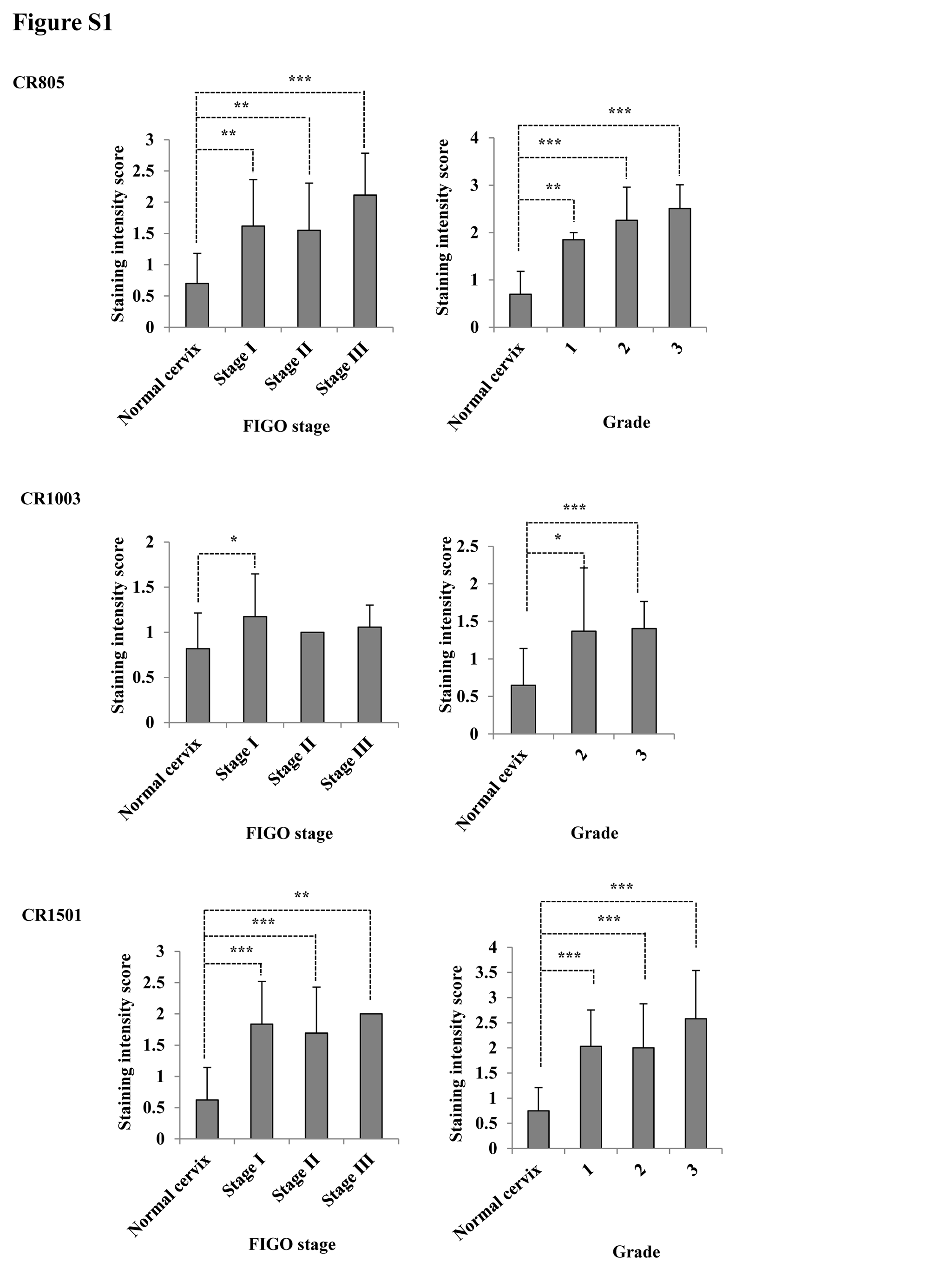

Supplement: Figure S1 — The expression of TACC3 in cervical cancer with respect to stage of the disease and histological grading. The expression of TACC3 with different disease stages and tumor grade was presented. *, p<0.05; **, p<0.01; ***, p<0.001 (TIF) [file pone.0070353.s001.tif]

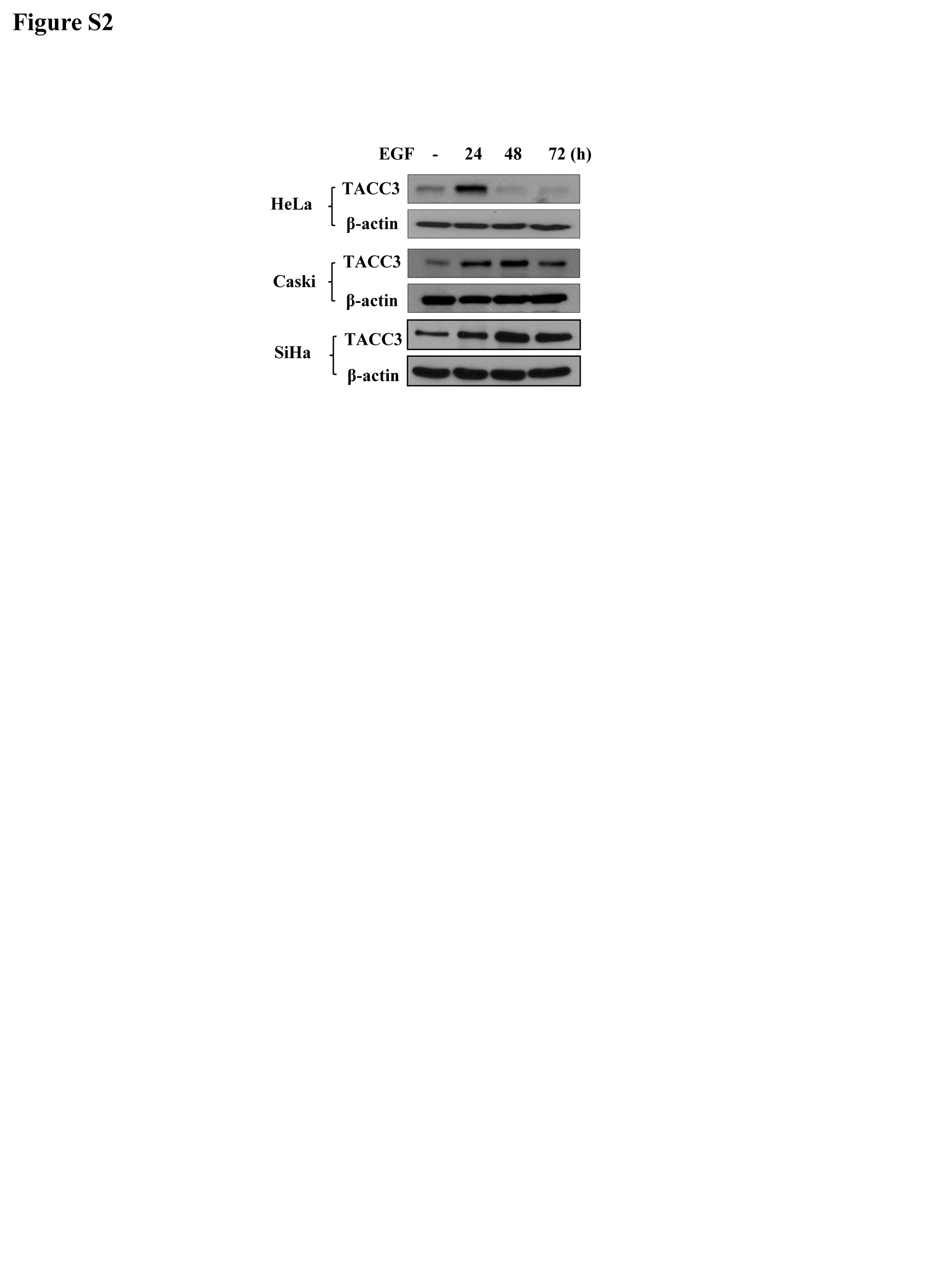

Supplement: Figure S2 — EGF stimulation induces endogenous TACC3 expression. TACC3 was induced by EGF treatment. Cells were incubated with or without 50 ng/ml and then collected at the indicated time points for western blot analysis. β-actin was used as a loading control. (TIF) [file pone.0070353.s002.tif]
